# Transplant center practices that maximize access to living kidney transplantation: the Penn experience

**DOI:** 10.3389/fimmu.2025.1700930

**Published:** 2026-01-20

**Authors:** Emre Arpali, Samir Abu-Gazala, Robert R. Redfield, Amanda Leonberg-Yoo, Ty B. Dunn

**Affiliations:** 1 Department of Surgery, Medical College of Wisconsin, Milwaukee, WI, United States; 2 Department of Surgery, Hospital of the University of Pennsylvania, Philadelphia, PA, United States; 3 Department of Surgery, University of California Irvine, Irvine, CA, United States; 4 Department of Medicine, Hospital of the University of Pennsylvania, Philadelphia, PA, United States

**Keywords:** donor engagement, donor navigator, financial support, kidney paired donation, living donor kidney transplantation, microsite outreach, transplant access, voucher program

## Abstract

Living donor kidney transplantation (LDKT) provides superior outcomes for patients with end-stage kidney disease (ESKD) but remains underutilized due to persistent barriers including donor-recipient incompatibility, financial concerns, and limited donor outreach. This study describes a center-based strategy implemented at Penn Medicine’s Center for Living Donation to specifically address these challenges and expand access to LDKT. Key components included the development of a dedicated infrastructure for donor evaluation, the integration of Living Donor Navigators to guide and support donors, and the use of personalized microsites through the National Kidney Registry (NKR) to facilitate recipient-driven outreach. Financial barriers were reduced through accessing national assistance programs, while participation in kidney paired donation and the NKR Voucher Program enabled flexibility in overcoming immunologic and timing obstacles. This coordinated approach resulted in a substantial increase in living donor transplants, including more than 100 KPD transplants in a single year—a global record. Donor and recipient satisfaction was consistently high, underscoring the effectiveness of the center’s patient-centered model. These findings demonstrate the impact of structured and scalable interventions designed to enhance access to LDKT. The Penn model offers a practical framework for other transplant centers seeking to reduce barriers and expand living kidney donation.

## Introduction

End-stage kidney disease (ESKD) is a significant public health challenge, with kidney transplantation recognized as the optimal treatment modality. Living donor kidney transplantation (LDKT) offers superior outcomes compared to deceased donor kidney transplantation, including faster access to transplant, better short- and long-term graft function, and longer patient survival. Despite these advantages, numerous barriers—such as immunologic incompatibility, financial constraints, limited donor awareness, and country-specific transplant infrastructure issues—impede the increased implementation of LDKT that is necessary to mitigate the shortage of kidneys available for transplantation (1, 2).

A multifaceted approach was designed to increase living donor kidney transplantation and to address pervasive challenges existing in the field. This article reviews the specific strategies and the results of their implementation at a single center. This model includes components that may be replicable in other transplant centers and other countries and has the potential to address disparities in underrepresented populations and to grow access to living donor kidney transplantation on a greater scale (3–5).

## Methods

**Figure 1:**
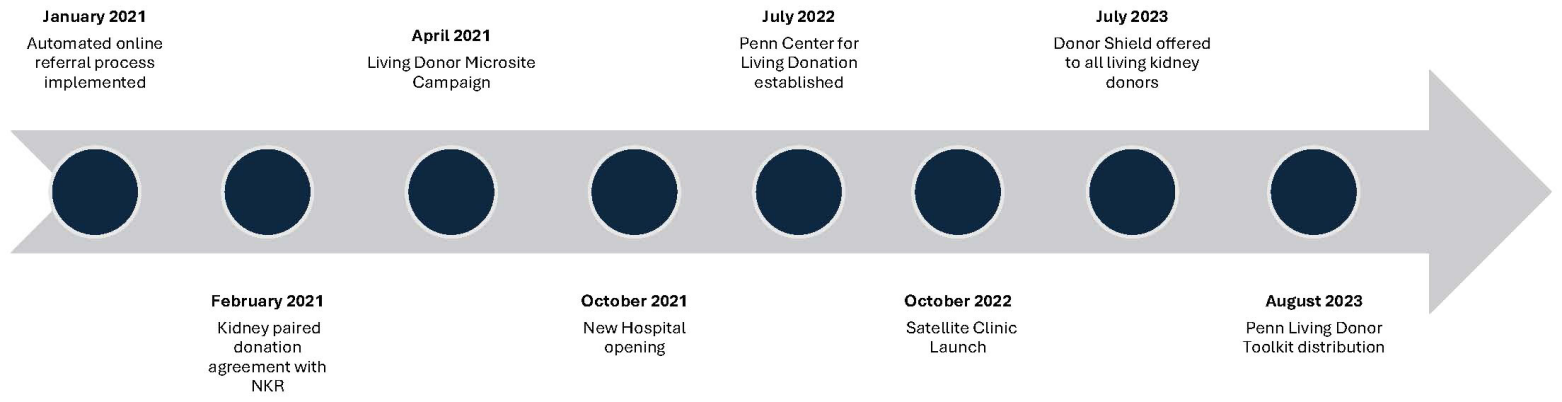
Timeline of programmatic events and implementation of initiatives that increased support of living kidney donation at Penn Medicine.

**Figure 2:**
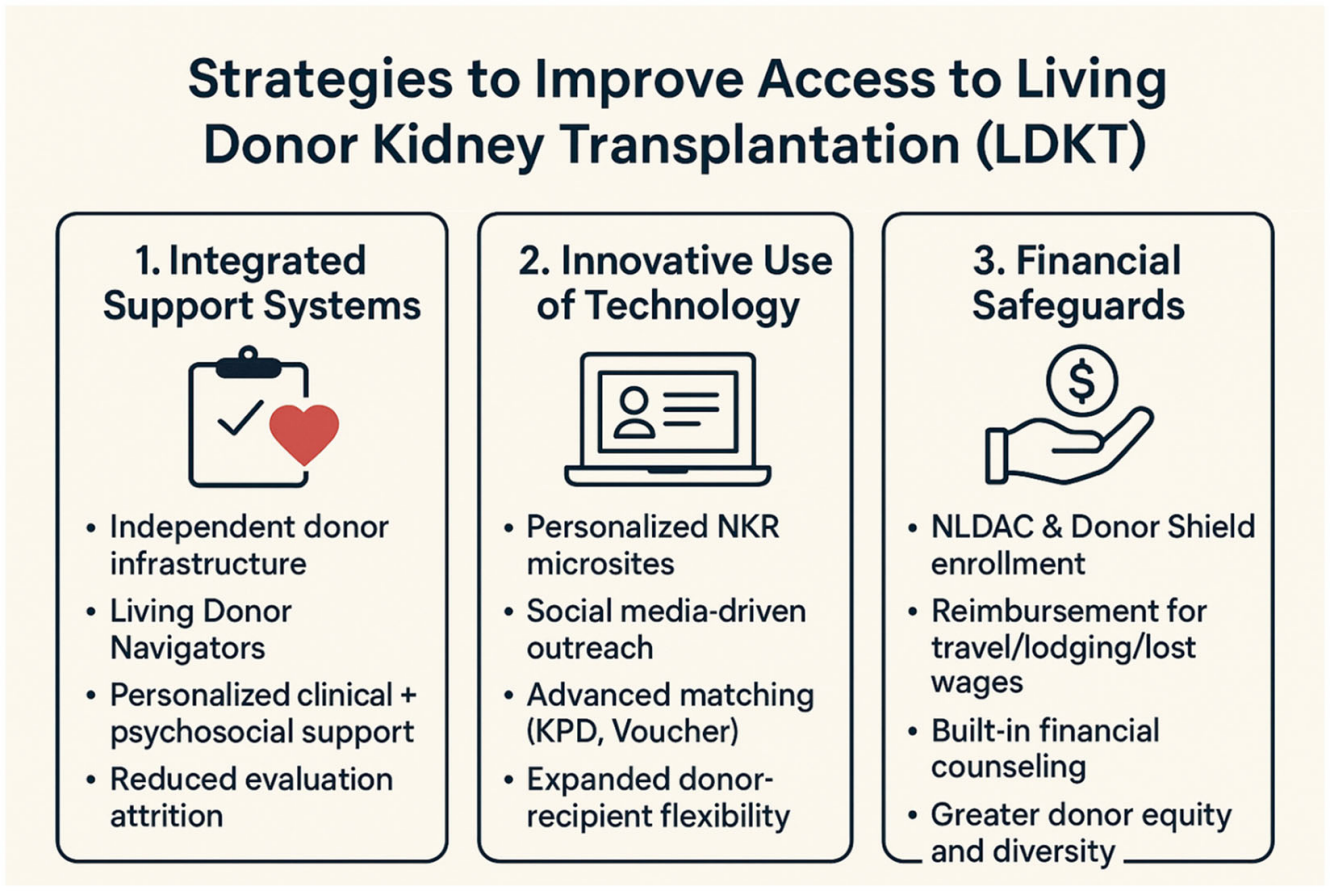
Strategies designed to enhance the experience of potential living kidney donors included innovative support systems, integrated use of technology, and financial safeguards. These strategies were targeted for implantation at the center level, patient level, and system level.

Our center developed and implemented specific methods to support potential living donors and their recipients in their donation or transplant journey, beginning in 2021 (**Figure 1**). Initiatives were multipronged and leveraged at the center level, patient level, and system level, and are summarized by **Figure 2**. This work reports a retrospective study of programmatic quality improvements and their impact on access to care and program growth. Program-level activity data was extracted from our internal database, the National Kidney Registry, and the National Living Donor Assistance Center. Chi-square analysis of categorial values was done using GraphPad Prism version 10.1.1 (Boston, MA) with statistical significance set at p=<0.05.

### Center level initiatives

The Penn Medicine Center for Living Donation was established in July 2022 as a result of a strategic initiative to grow the living donor programs (kidney, liver, uterus) while providing a comprehensive infrastructure to support exceptional care for the potential living donor. The Center is supported by dedicated administrative and clinical resources. In addition to the core team members (surgeons, medical subspecialists, physician assistants and social workers, and an independent donor advocate) specializing in the care of living donors, we added dedicated administrative support including a Living Donor Manager and a finance coordinator who oversee the program [a total of 1.5 full time equivalent (FTE)]. An additional 3.0 FTEs in clinical resources were hired, including a referral intake coordinator, and specialized living donor and recipient coordinators. To support the desired patient experience and to support the expansion of clinic sites, a Patient Navigator was hired (1.0 FTE). This team was built to ensure streamlined operations throughout the continuum of care—from initial referral to long-term post-donation follow-up—and functions independently from the transplant recipient organ programs, thereby reducing potential conflicts of interest and maintaining donor-focused priorities. Resource-based multidisciplinary clinics were established to accommodate evaluation sessions on multiple days of the week, with team meetings occurring on a weekly basis. The kidney donor program implemented a dedicated quality assessment and performance improvement (QAPI) dashboard and morbidity conference, which convened on a quarterly basis.

### Patient level initiatives

**Figure 3:**
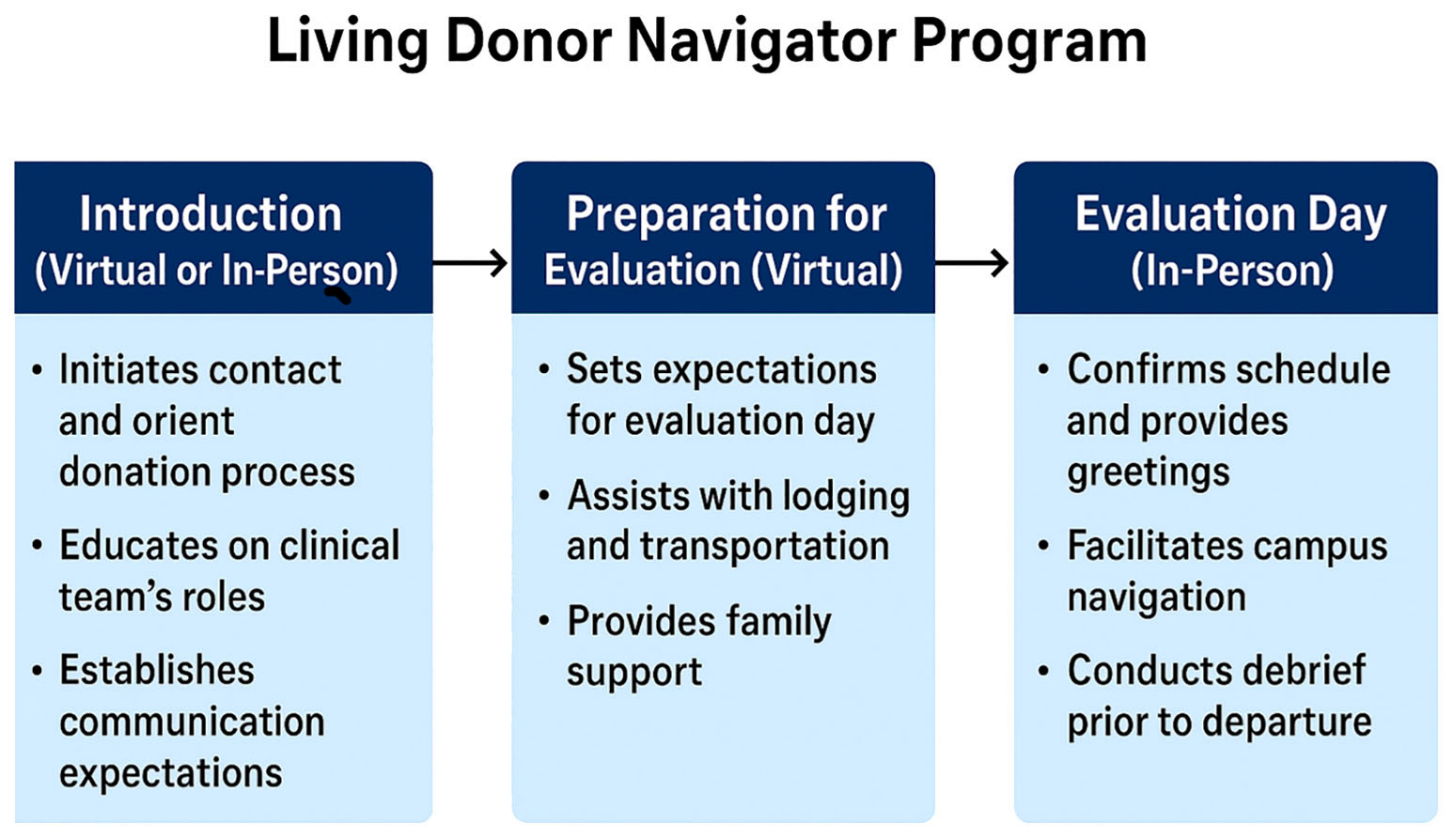
Phases of the living donor navigator workflow.

A central innovation within the center’s infrastructure is the Living Donor Navigator Program, which provides individualized support to donor candidates and their families. Navigators are trained medical or non-medical personnel with in-depth knowledge of the donation process (6). They offer education, emotional guidance, and logistical coordination, including assistance with scheduling, lodging, transportation, and follow-up care in a culturally sensitive fashion. Their involvement enhances the donor experience, reduces barriers to evaluation, and promotes continuity and clarity throughout the donation journey. The Living Donor Navigator Program was designed to support potential donors of all ethnicities across three structured phases (**Figure 3**).

#### Introduction

The navigator initiates contact to explain their role and orient the donor to the transplant process. This includes education on the clinical team’s responsibilities and setting expectations for communication and engagement throughout the evaluation. This phase can be offered virtually or in person.

#### Preparation for evaluation

Navigators provide detailed guidance about the upcoming evaluation day, communicate the schedule in a geographically and logistically considerate manner, and help the donor prepare for non-clinical challenges, such as lodging, transportation, parking, and family accommodations. This phase is performed virtually.

#### Evaluation day

On the day of evaluation, navigators offer in-person support by welcoming the donor, confirming schedules, facilitating special services (e.g., language interpretation), and guiding them through campus logistics. A structured debrief is conducted at the end of the evaluation day to reinforce next steps and address any remaining concerns.

This phased, patient-centered approach exemplifies Penn’s commitment to accessibility, empathy, and operational excellence - key programmatic features that contributed to a record breaking number of living donor transplants achieved through kidney paired donation (7).

### System-level initiatives to expand access

Despite the clear advantages of LDKT, a significant proportion of transplant candidates lack an identified potential living donor. Many individuals do not have effective avenues or comfort level needed to communicate their need for a kidney transplant, resulting in missed opportunities for timely transplantation. To address this challenge, our center has adopted several system-level strategies aimed at expanding access to LDKT through outreach, logistical support, and innovative matching solutions.

#### Recipient outreach via social media and NKR microsites

To increase donor referrals, we partner with the National Kidney Registry (NKR) to offer personalized microsites which are free to patients. These are small, standalone websites designed specifically for transplant candidates (8). These microsites empower recipients to share their personal stories and express their need for a kidney in an accessible and emotionally compelling format. Designed for easy dissemination via social media, text, and email, the microsites facilitate broad outreach within the recipient’s social network. Each microsite also includes a confidential link that allows potential donors to discreetly begin the referral process. This patient-driven digital engagement approach reduces psychological and logistical barriers, improves visibility, and increases the likelihood of donor referrals.

#### Financial protection programs for donors

Acknowledging the financial burdens often associated with donation, donors at our center can access national programs such as the National Living Donor Assistance Center (NLDAC) and the NKR Donor Shield (9, 10). The NLDAC is available to lawfully residing residents and citizens of the United States who are potential living donors working with any program in the United States when their expenses cannot be reimbursed by the recipient, an insurance company, or a state program. The program is administered by the Division of Transplantation, Healthcare Systems Bureau, Health Resources and Service Administration, and the United States Health and Human Services (HHS) through a cooperative agreement with the University of Kansas and the American Society of Transplant Surgeons. Eligibility depends on the transplant recipient’s household income being less than 350% of the HHS poverty guideline. Year over year NLDAC applications and financial support granted to Penn Donors is summarized in **Table 1**. Donor Shield is available to potential donors via the NKR if they facilitate a NKR donation or donate at a Donor Shield Direct center, and support is not contingent on recipient household income. This program provide reimbursement for travel, lodging, dependent care, and lost wages, and coverage for complications. Both programs reduce economic disincentives and make donation more accessible across diverse socioeconomic backgrounds.

**Table 1:** NLDAC applications and financial assistance provided to Penn living donors.

NLDAC Applications Summary						
Year	Applications	Approved Applications	% Approved Applications	Surgery Completed	% Surgery Completed	NLDAC Financial Support
2008	5	5	100%	4	80%	$19,746.45
2009	6	6	100%	4	67%	$8,014.19
2010	4	4	100%	3	75%	$4,463.56
2011	7	5	71%	3	60%	$4,358.58
2012	8	7	88%	4	57%	$12,130.88
2013	5	3	60%	3	100%	$5,818.86
2014	8	6	75%	5	83%	$8,534.03
2015	9	8	89%	4	50%	$20,704.54
2016	10	6	60%	5	83%	$16,738.93
2017	9	4	44%	3	75%	$11,963.84
2018	4	4	100%	2	50%	$7,718.91
2019	11	8	73%	4	50%	$15,075.86
2020	6	4	67%	1	25%	$4,369.70
2021	15	12	80%	6	50%	$24,576.59
2022	21	14	67%	14	100%	$44,437.33
2023	8	6	75%	6	100%	$15,046.72

#### Kidney paired donation and voucher programs

To address immunologic incompatibility and other logistical challenges, we actively participate in Kidney Paired Donation (KPD). This allows donor-recipient pairs to be matched with others, facilitating transplants that would otherwise not be possible. There are several regional and national KPD programs in the United States, with many participating transplant centers. The NKR is the largest program, with 102 participating centers. Their development of voucher programs has facilitated new opportunities for LDKT (11). A standard voucher provides a mechanism for advance donation where the intended recipient receives a voucher for a living donor kidney. This added flexibility accommodates the schedules and readiness of both donors and recipients, further expanding the donor pool and improving transplant timing. A family voucher is a mechanism for altruistic donors that provides a voucher for up to 5 named individuals (with one allowable redemption) in the event a loved one needs a kidney transplant in the future. Both standard and family voucher donors are also personally protected by being prioritized to receive a kidney from the NKR should the donor ever need one. Together, these system-level interventions form a comprehensive framework that improves access, reduces barriers, and supports patients and donors throughout the LDKT process.

## Results

The implementation of system-level strategies at Penn Medicine has resulted in substantial improvements in access to LDKT. These efforts have produced sustained growth in donor engagement, transplant volume, and institutional visibility, which reflect the success of Penn’s integrated and patient-centered approach.

### Microsite utilization and impact

Since their introduction in 2021, personalized microsites have shown consistent and accelerating adoption. In 2025, 48% of patients on Penn’s waitlist have an active microsite. As more patients leveraged this platform to share their transplant story, microsite-generated referrals steadily translated into completed living donor transplants. Among patients with active microsites at Penn, 28% received living donor transplants in 2025. This trend underscores the value of digital, recipient-driven outreach in identifying and engaging potential donors.

### Rising living donor transplant volumes

**Figure 4:**
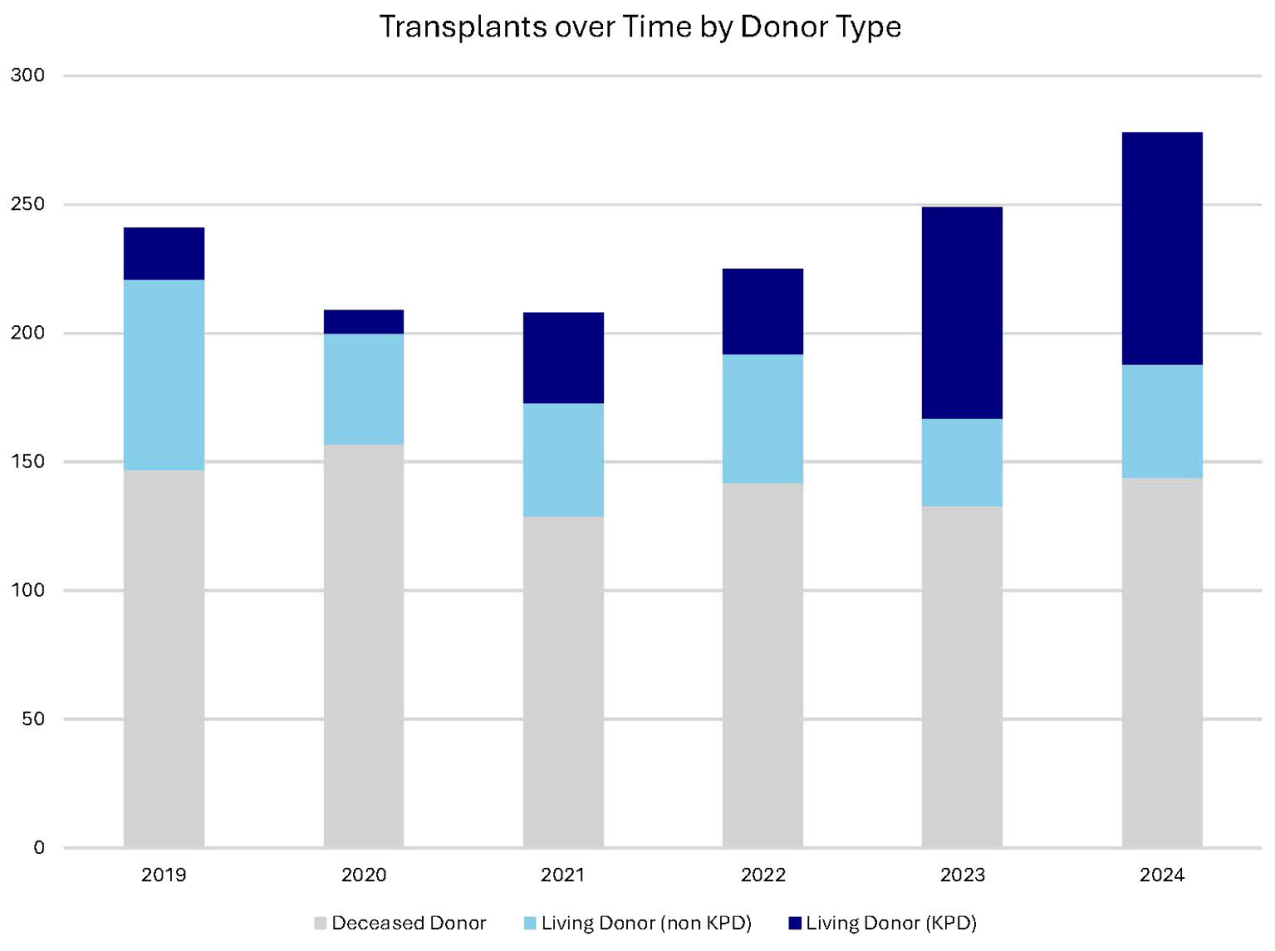
Kidney transplant activity at Penn by year and donor type, illustrating the growth of the program during the timeline of programmatic enhancements.

Penn has experienced a marked year-over-year increase in living donor kidney transplant activity (**Figure 4**). This growth aligns with the launch of key programmatic enhancements—including the establishment of the Center for Living Donation, integration of donor navigators, and expansion of outreach and financial support services—demonstrating the cumulative impact of coordinated, system-level investment.

### Enhanced donor and recipient experience

High levels of satisfaction have been consistently reported by both donors and recipients. The NKR Donor Satisfaction Survey results of donor scoring of their evaluation, pre-operative and donation processes have exceeded 4.8 out of 5 stars, and survey response volume nearly doubled after patient navigator integration in 2022. Patients cite personalized support, transparent communication, and logistical coordination as key contributors to their positive experience. Comparison of the cohorts from 2017-2019 (pre-resources) and 2021-2023 (post-resources) showed a significant increase in non-white transplant recipients (21% vs 33%, p=0.003). In addition, qualitative feedback from healthcare providers has cited improved support, efficiency, and relief of burnout and stress related to these system level interventions. These outcomes reflect the value of dedicated navigators, educational resources, and financial protections embedded within the program.

## Discussion

The experience at Penn Medicine illustrates how a comprehensive, multi-level strategy can significantly improve access to LDKT and result in programmatic growth. By investing in infrastructure in a targeted fashion, embedding patient-centered innovations, and deploying wide-reaching outreach mechanisms, the program achieved record transplant volumes and consistently high levels of donor and recipient satisfaction. This facility-based financial investment was a key driver that allowed maturation of the program. These outcomes are not unique to Penn’s institutional setting; rather, the strategies employed are intentionally designed to be adaptable and scalable, offering a practical blueprint for other transplant centers aiming to expand access to LDKT. While financial, logistical, cultural, and regulatory challenges differ across programs and countries, innovation should be multifaceted, tailored to the local environment and always focused on donor safety and financial neutrality.

A cornerstone of the program’s success was the establishment of a dedicated infrastructure for living donor care, which is operationally distinct from the recipient transplant program. This separation mitigated potential conflicts of interest and enabled staff to maintain an exclusive focus on donor needs. The integration of Living Donor Navigators further strengthened the donor-centered framework. This structured, individualized guidance helped reduce evaluation attrition, streamline workflows, and enhance donor satisfaction. We specifically leveraged KPD to prioritize the donor’s needs and separate them from the recipient’s needs, which avoided difficulties that can occur due to differing goals in timing, blood or tissue type matching, or anatomic considerations. These elements may be replicated in part or in whole within other settings with varying resource levels, allowing institutions to reconfigure existing staffing models to better support donor navigation and coordination. A donor-centric program may offer unique advantages to patients with anatomic issues (nutcracker syndrome, renal artery aneurysm) and otherwise normal renal function who may choose altruistic donation over other surgical solutions.

Digital engagement played a transformative role in expanding donor outreach and optimizing recipient matching. Personalized microsites provided an avenue for recipients to share their stories in an emotionally compelling and widely accessible format. This approach empowered patients and their social networks to take an active role in donor identification, thereby lowering traditional psychological and logistical barriers. In parallel, our robust participation in KPD and NKR-enabled Voucher programs created flexibility around compatibility and donation timing. These technology-driven tools are readily available to other centers in the United States and can be integrated into existing workflows with appropriate institutional buy-in and patient education.

We acknowledge there are limitations in transferability, especially in environments without current infrastructure for a strong national exchange program, however learnings from our single center organizational approach can be selectively applied, recognizing that center-level biases may impact outcomes.

Economic disincentives remain a persistent deterrent to living donation (2). Penn addressed this barrier head-on by embedding financial counseling and support into the evaluation process and ensuring that eligible donors accessed programs such as the National Living Donor Assistance Center (NLDAC) and Donor Shield. These resources cover essential costs like travel, lodging, and lost wages, thereby reducing disparities and improving donor diversity. Importantly, these financial supports are accessible at transplant centers nationwide and require only modest institutional coordination for implementation, making this aspect of the Penn model highly transferable in the United States and in other countries that can invest in a similar resource. Looking ahead, we are exploring the integration of telehealth to facilitate donor evaluation and reduce geographic barriers. Virtual consultations have the potential to expedite the workup process and ease logistical burdens, especially for donors living far from transplant centers. The program also plans to expand outreach in underserved communities by partnering with local organizations and tailoring education efforts to improve health literacy and trust. The donor-centered approach and administrative framework of the Center for Living Donation is also poised to actualize extra-renal and trans-organ exchanges given the expertise of our living donor liver and kidney programs (12, 13).

The Penn model provides a flexible, resource-informed framework that can be adapted across a range of institutional contexts—from high-volume academic centers to smaller regional programs. The success of this approach lies not in any single innovation, but in the deliberate layering of multiple strategies—each reinforcing the others to create a cohesive, donor-centered system. By adopting and tailoring these elements to meet their own operational and community needs, other centers can build locally appropriate models to improve LDKT access. Features of this model may also inform national and international initiatives aimed at reducing disparities and increasing access to living donor transplantation.

## Data Availability

The raw data supporting the conclusions of this article will be made available by the authors, without undue reservation.
